# The relationship between anxiety, productivity and self-compassion of healthcare professionals in COVID19 time

**DOI:** 10.1192/j.eurpsy.2023.1693

**Published:** 2023-07-19

**Authors:** M. Cornea, C. Bacila

**Affiliations:** 1 Clinical Hospital of Psychiatry, Dr. Gheorghe Preda” Sibiu; 2 Faculty of Medicine, Lucian Blaga University, Sibiu, Romania

## Abstract

**Introduction:**

During the Corona virus pandemic, anxiety was one of the most felt emotions. Anxiety can lead to maladaptive coping behaviors, including decreased productivity at work. There are some mechanisms that buffer the devastaing effect that anxiety can have, and one of them is self-compassion. The impact on mental health during the pandemic has increased, especially among healthcare professionals, and needs further exploration

**Objectives:**

This study aims to investigate the relationships between anxiety, productivity and self-compassion at work among healthcare professionals, as well as, the possibility of self-compassion playing the role of a relationship’s moderator, in a pandemic context.

**Methods:**

The study gathers the result from 202 participants, who completed a questionnaire on an online platform. We examined the link between anxiety, respectively COVID-19 anxiety, productivity and self-compassion, as well as the link between self-compassion and productivity among healthcare workers in a pandemic context. In order to emphasize the objectives, the validated BAI, CAS, SPS-SV and SCS-SV scales were used.

**Results:**

In terms of results, significant negative correlations were identified in the relationships between anxiety, COVID-19 anxiety, productivity and self-compassion, and a significantly positive correlation in the self-compassion-productivity relationship. Also, we studied if self-compassion could be a relationship moderator. As the results show, the study identified a statistically insignificant effect of self-compassion on the relationships anxiety-productivity and anxiety of COVID-19-productivity, among health workers.

**Conclusions:**

The present study has achieved its proposed objectives, so that through future research, anxiety, productivity and self-compassion can be explored not only in a pandemic context, but also in a normal context of professional activity in the medical field, and it can also contribute to the identification of other moderators of the anxiety-productivity relationship.

**Disclosure of Interest:**

None Declared

